# The impact of intergenerational transmission of stigma on the psychological development of minors with HIV-positive parents

**DOI:** 10.3389/fpsyg.2026.1911490

**Published:** 2026-09-18

**Authors:** Chenxi Li, Jiali Zhang, Runze Gao, Lin Cai, Tongtong Yang, Ye Wang, Fan Luo

**Affiliations:** 1 Department of Radiology, Chendu Public Health Clinical Medical Center, Chendu, China; 2 Department of Psychiatry and Psychology, Beidaihe Rest and Recuperation Center, Qinhuangdao, Hebei, China; 3 Department No.2 of Convalescence, Beidaihe Rest and Recuperation Center, Qinhuangdao, Hebei, China; 4 Department No.2 of Infectious Disease Unit, Chendu Public Health Clinical Medical Center, Chendu, China; 5 Department No.1 of Infectious Disease Unit Chendu Public Health Clinical Medical Center, Chendu, China

**Keywords:** family system, HIV-positive parents, intergenerational transmission, minor children, stigma

## Abstract

With the widespread adoption of antiretroviral therapy, HIV infection has become a chronic, manageable condition, and the population of HIV-positive parents continues to grow. The stigma linked to their condition passes down to their minor children within the family. This harms the children’s mental health and makes it harder to prevent and control HIV. This review explores the patterns and mechanisms of intergenerational transmission. It uses stigma theory and ecological systems theory. By examining evidence from around the world and within China, it highlights transmission features and how to adapt interventions specifically for the Chinese context. The results indicate that the stigma experienced by HIV-positive parents exhibits three-dimensional overlap, role specificity, and chronic persistence, with significant population heterogeneity. Intergenerational transmission happens through a four-level chain. It includes family communication and disclosure, parent–child attachment, and family functioning. Next, parental emotions and behaviors come into play. Finally, children’s understanding and internalisation of stigma follow. This process shows a dose–response relationship with psychological harm in children. Among existing interventions, family-system-integrated interventions yield the best outcomes, supplemented by individual parental interventions, child protection measures, and social structural interventions; these must be adapted to local cultural and population characteristics. The study shows that we can intervene in the intergenerational passing of disease-related shame. Future research should include longitudinal studies, focus on specific populations, and develop multi-system collaborative intervention models to safeguard the rights of affected children and improve the comprehensive HIV/AIDS prevention and control system.

## 1 Introduction

Stigma, as a classic sociological and psychological construct, refers to the process by which individuals or groups are labelled, ostracised, or discriminated against on the basis of attributes that are socially devalued. Goffman famously defined it as “an attribute that is deeply discrediting,” (Goffman, 2009) while Scambler further distinguished between “enacted stigma” and “felt stigma,” emphasising that health-related stigma is not merely a product of social structures but also profoundly shapes individuals’ psychological well-being and behavioral responses (Scambler, 2009). Stigmatisation in infectious diseases can be more harmful than the disease itself. It does not just affect the person with the illness; it spreads through families and communities. This creates lasting psychological effects that can impact future generations (Ranwa and Arora, 2025; Bharat, 2011). This creates lasting psychological effects that can impact future generations. HIV-related stigma is one of the most harmful types of health stigma. It is closely linked to sensitive topics like sexuality, drug use, and moral judgement (Brown et al., 2010; Jones and Corrigan, 2014).

With the widespread adoption of antiretroviral therapy (ART), HIV infection has evolved into a chronic infectious disease that can be managed long-term, and the population of HIV-positive parents continues to grow both globally and in China (Zerdali et al., 2023; Mchenry et al., 2019). As of 2023, about 38.4 million people live with HIV globally. Over 15 million of these are adults of childbearing age. Each year, around 2.3 million children are newly affected. In China, 1.204 million people have HIV. Of these, 30% are parents with minor children. The epidemic mainly affects the Southwest and Central South regions, as well as areas with high ethnic minority populations. HIV-related stigma combines negative social stereotypes and internal shame (Goffman, 2009). It is a key barrier that affects family functioning in this group. It is transmitted intergenerationally through the family system, creating a chain reaction: “parental stigma → distorted family environment → psychological harm to children” (Qiao et al., 2015; Ashry et al., 2017).

Evidence-based research confirms that the prevalence of anxiety and depression symptoms among children of HIV-positive parents is significantly higher than that of the general pediatric population (Visser et al., 2018; Pelton et al., 2021), and their health-related quality of life exhibits marked deficits in psychological and social dimensions (Pelton et al., 2021). This intergenerational transmission not only directly threatens the mental health of children and adolescents and exacerbates their difficulties in social adaptation in adulthood, but also undermines parental treatment adherence and children’s understanding of HIV prevention and control, thereby indirectly hindering the advancement of HIV/AIDS prevention and control efforts (Zerdali et al., 2023; Qiao et al., 2015). So, it indirectly slows down efforts to prevent and control HIV/AIDS. So, clearly explaining how HIV/AIDS spreads and how to intervene is crucial. This work is urgent to protect affected children’s rights and interests. It also plays a key role in enhancing the overall prevention and control system for HIV/AIDS.

Existing research has made substantial progress in understanding HIV-related stigma among parents. First, studies show its four-dimensional structure: enacted, felt, anticipated, and internalized stigma. They also highlight how it varies by role in the parental context (Goffman, 2009; Qiao et al., 2015). Second, strong evidence links parental stigma to issues in children’s emotional, cognitive, social, and behavioral development (Visser et al., 2018; Das et al., 2017). Third, family-based intervention models are proven to be feasible (Rochat et al., 2016; Betancourt et al., 2017). Despite these advances, gaps still exist. Most intervention studies are small and short-term. They do not validate effects across regions, ethnic groups, or family structures and lack long-term follow-up (Wang, 2021; Chu and Wenbo, 2014). Also, there’s little research on localization and adaptability. Studies often ignore local factors like traditional Chinese culture and structural discrimination (Betancourt et al., 2017; Xing, 2020). Finally, the dose–response relationship between parental stigma and child harm is not clear. The limits of protective factors are still uncertain too (Rzeszutek et al., 2021; Chi and Li, 2013). These gaps make it difficult for existing research to support the development of precise intervention strategies.

This review combines evidence from both domestic and international sources. It aims to clarify the features and mechanisms of intergenerational stigma transmission among HIV-positive parents. It looks at how effective interventions are and how these ideas fit various populations. Special attention is given to cultural specifics and structural dynamics in China. The goal is to fill existing gaps in our understanding. The core objectives include: mapping the characteristics of stigma and the phenotypic manifestations of psychological harm in children; analyzing the mediating pathways and moderating factors of transmission and constructing an integrated model; and synthesizing evidence on intervention effectiveness while proposing recommendations for localized adaptation.

## 2 Methodology

This systematic review followed the Preferred Reporting Items for Systematic Reviews and Meta-Analyses (PRISMA) 2020 guidelines. We searched PubMed, Web of Science Core Collection, PsycINFO, and China National Knowledge Infrastructure (CNKI) for literature published between January 2009 and April 2026, using a combination of Medical Subject Headings (MeSH) terms and free-text words covering HIV/AIDS, stigma, intergenerational transmission, family, child psychological development, and interventions. We also manually screened the reference lists of included studies.

We used the Population, Intervention, Comparison, Outcomes, and Study Design (PICOS) framework to include studies on HIV-positive parents and their minor children (under 18 years). These studies needed to report at least one quantitative outcome. This could be about parental stigma, child mental health, or family functioning. Mechanistic studies could use cross-sectional, cohort, or case–control designs. However, intervention studies must include a control group, like a randomized controlled trial (RCT) or a quasi-experimental design. We excluded duplicates, non-original publications, studies without a parental role or child data, and those that did not separately distinguish minor children or HIV positive parent populations.

Two reviewers independently screened the initial 1,027 records (367 from PubMed, 450 from Web of Science, 160 from PsycINFO, and 50 from CNKI), ultimately retaining 73 articles. We used a standard form to gather study details, participant information, effect sizes, and mediators for mechanistic studies. We also collected intervention components and outcome measures, checking everything to ensure accuracy.

For quality appraisal, we used RoB 2.0 for RCTs, the Newcastle Ottawa Scale for cohort and case–control studies, and the National Institutes of Health (NIH) tools for cross-sectional studies. Disagreements were resolved through discussion or consultation with the corresponding author. Given the considerable heterogeneity across designs, populations, interventions, and outcomes, we adopted a narrative synthesis. Mechanistic evidence was organized along a fourtier mediating pathway—family communication and disclosure, parent child attachment and family functioning, parental emotion and parenting behaviors, and children’s cognition and stigma internalization. Intervention studies were classified into four types: parent-targeted individual, family based integrated, child protective, and socio ecological structural interventions. Where possible, we extracted and compared effect sizes, and graded the overall evidence quality using the Grading of Recommendations, Assessment, Development and Evaluations (GRADE) system.2 Characteristics of Stigma Among HIV-Positive Parents and Phenotypic Patterns of Impaired Psychological Development in Their Children.

### 2.1 Core characteristics of stigma among HIV-positive parents and population heterogeneity

HIV-related stigma is a complex issue. It comes from society’s negative stereotypes and discrimination against people with HIV. It also involves individuals feeling shame and denying their situation. Its core essence lies in a chain reaction involving “social labeling—self-identity—behavioral adaptation” (Lian et al., 2022). Among HIV-positive parents, disease-related stigma is not a unidimensional psychological experience. Rather, it constitutes a multi-layered cognitive-emotional-behavioral system formed by superimposing the specificity of the parental role onto the classic four-dimensional structure of public stigma, experienced stigma, anticipated stigma, and internalized stigma. Furthermore, it exhibits significant heterogeneity across different subgroups.

#### 2.1.1 Core characteristics

Disease-related shame in HIV-positive parents has three key features: it appears in layers, relates to their specific roles, and tends to last over time. Three-dimensional superposition is characterized by the intertwining of structural social stigma, individual internalized stigma, and family system stigma: discriminatory policies at the societal level and community exclusion form the foundation of structural stigma (Aghaei et al., 2023); parents internalize this external rejection as self-shame and a negative self-image, forming the core of internalized stigma (Rzeszutek et al., 2021); and the family, as a vehicle for transmitting disease-related shame, further generates family system stigma centered on “secrecy” and “avoidance,” with all three factors collectively amplifying the intergenerational impact on children (Kennedy et al., 2010). Role specificity is a key feature of disease-related shame in parents. It shows up mainly as worry about “courtesy stigma” towards their children (Ma et al., 2019). It shows up mainly as worry about “courtesy stigma” towards their children. Parents with HIV feel shame about their own infection. They also worry their children might be excluded socially because of the family’s status. This concern boosts their hiding behaviors and social isolation. It becomes a key factor in passing down disease-related stigma across generations (Nabunya and Namuwonge, 2023). Chronicity stems from the chronic nature of HIV infection and the persistence of social stigma. The disease-related stigma experienced by HIV-positive parents is not an acute stress response but a chronic psychological state that accompanies the entire disease management process. Structural discrimination in society keeps this experience alive. It makes disease-related stigma a long-term risk for families (Sroufe, 2013).

#### 2.1.2 Population heterogeneity

There are notable differences in disease-related shame among HIV-positive parents. These variations appear mainly in two areas: First, gender plays a significant role in how stigma affects these parents. Female HIV-positive parents feel more internalized stigma than males. Their shame about the disease often mixes with guilt over parenting duties and pressure from traditional gender roles. They also worry more about how this stigma might impact their children (Zhang, 2011). Female HIV-positive individuals face more fear about disclosing their status than men. They also tend to have a more negative self-image. To deal with stigma, they often choose to hide their status or avoid situations altogether (Operario et al., 2022). Second, the route of HIV transmission and the presence of overlapping stigmatized identities further differentiate stigma experiences. Parents infected by men who have sex with men (MSM) deal with two types of stigma: being part of a sexual minority and having HIV. This combined stigma raises their overall feelings of shame. As a result, they often face greater social isolation and find it harder to communicate with their families (Operario et al., 2022). In contrast, parents infected through blood or mother-to-child transmission experience shame primarily focused on fear of the disease itself, with relatively weaker moral self-blame (Liu et al., 2021). Third, socioeconomic status and age generate additional variation. Parents with lower education, those in rural areas, and low-income families face more structural stigma. They feel greater disease-related shame and have fewer coping resources (Xiong et al., 2022). For middle-aged and older parents, shame often connects to failing family duties and worries about reputation. In contrast, younger parents focus more on the risk of spreading disease during childcare and fear of social discrimination (Anderson et al., 2015). Fourth, geographical and cultural contexts shape stigma experiences in distinct ways. In areas with high HIV rates, like the Liangshan Yi Autonomous Prefecture in southwestern China, parents deal with greater community stigma and discrimination. This often leads them to choose family-wide social isolation strategies (Nabunya et al., 2023). In these settings, the close-knit kinship networks and community ties that ordinarily provide social support paradoxically amplify the effect of “courtesy stigma,” leading families to opt for complete concealment out of concern for the extended family’s reputation. Ethnic customs about illness and death make it harder for parents to handle their HIV status. This often turns external discrimination into family secrets. Urban parents have better access to healthcare, but they worry more about their infection status affecting their children’s education and school admission. Their concerns often stem from fears of losing social mobility (Sroufe, 2013). These differences between urban and rural areas, as well as ethnic groups, show that interventions should consider local cultural norms and available resources. A one-size-fits-all approach will not work.

It is also worth noting that the geographical distribution of the included studies is uneven. Of the 45 articles ultimately retained, 17 (37.8%) came from China, 11 (24.4%) from sub-Saharan Africa, 8 (17.8%) from Europe and North America, 7 (15.6%) from South and Southeast Asia, and 2 (4.4%) from Latin America and other regions. Consequently, the conclusions of this review regarding the family communication pathway of intergenerational transmission and intervention adaptation predominantly reflect research evidence from Chinese and African settings, whereas studies from Western contexts tend to focus more on individual psychological traits and healthcare system responses. These differences between urban and rural areas, as well as ethnic groups, show that interventions should consider local cultural norms and available resources. A one-size-fits-all approach will not work.

### 2.2 Core phenotypes and stage-specificity of impaired psychological development in minors under intergenerational transmission of stigma

Intergenerational stigma affects children’s psychological growth in specific ways, not broadly. Its impact varies with age and focuses on four main areas: emotions, self-concept, social adaptation, and behavior development. Based on life-span theories in developmental psychology, critical windows in the psychological development of children and adolescents (the attachment formation period from 0 to 3 years, the self-concept development period from 6 to 12 years, and the identity formation period from 12 to 18 years) are more sensitive to family environmental stimuli. Consequently, the effects of intergenerational transmission of disease stigma also exhibit stage-specific differentiation (Abubakar et al., 2017), as shown in Table 1.

**Table 1:** Comparison of core phenotypes and stage specificity of impaired psychological development in minor children under intergenerational transmission of stigma dose–response relationship and population differences between parental stigma and children’s psychological harm.

Damaged dimension	Characteristics	Infancy (0–3 years)	Preschool period (3–6 years)	School age (6–12 years)	Adolescence (12–18 years)
Emotional dimension	High prevalence of negative emotions and deficits in emotion regulation	Labile mood, irritability and worsened separation anxiety	Generalized fear, frequent emotional outbursts, excessive anxiety over illness and death	Predominant depression and loneliness, extreme sensitivity to others’ evaluations	Rising comorbid emotional disorders, affective impulsivity, suicidal ideation
Self-perception dimension	Negative self-concept, identity confusion and low self-esteem	Cognitive immaturity	Delayed development of self-efficacy, lack of confidence in peer interaction	Development of self-negative cognition and stress from acting as the keeper of family secrets	Identity confusion, internalized affiliate stigma, poor self-worth
Social adaptation dimension	Deficient interpersonal trust, chronic social avoidance and poor peer relationships	Excessive vigilance toward strangers and surroundings, passive withdrawal	Low willingness for peer interaction and low participation in group play	Active avoidance of group activities and deliberate concealment of family conditions	Insular social network, impaired intimate bonding, social isolation
Behavioral dimension	High rates of internalizing/externalizing behavioral problems, academic and physical behavioral dysregulation	Hyperactivity, dysregulation of eating and sleeping behaviors	Aggressive behaviors, rule-breaking and prominent externalizing problems	Maladaptive academic adaptation (inattention, school truancy), somatic symptoms	Substance misuse, risky sexual conduct, antisocial acts, self-harm and suicidality

#### 2.2.1 Emotional dimension: high prevalence of negative emotions and deficits in emotional regulation

Impaired emotional functioning is the most common early sign in children. It shows a high rate of negative emotions like anxiety, depression, and loneliness. Also, these children often struggle with emotional regulation strategies (Das et al., 2017). Meta-analyses indicate that among HIV-affected children, the prevalence of depressive symptoms is 31.2%, and that of anxiety symptoms 28.7%. These rates are markedly higher than those observed in the general pediatric population. Furthermore, parental stigma has been identified as a core predictive factor (Visser et al., 2018). Stage-specific manifestations vary markedly across developmental periods. In infancy and early childhood (0–3 years), emotional instability, irritability, and heightened separation anxiety are central. Parents‘chronic negative emotions may diminish caregiving sensitivity, leading children to form insecure attachments and laying the groundwork for subsequent emotional difficulties (Mo et al., 2014). In preschoolers aged 3 to 6, it’s common to see general fear and emotional outbursts. They may also show a lot of anxiety about illness and death. Limited verbal skills can lead to behavioral problems as they express their distress (Peng, 2019). In the school-age period (6–12 years), depression and loneliness become the dominant phenotypes, accompanied by extreme sensitivity to others’ evaluations and prolonged durations of negative affect (Rodriguez et al., 2018). During adolescence (12–18 years), emotional disorders often increase significantly. This is linked to impulsivity in managing emotions. Sometimes, it can lead to ongoing low mood and thoughts of suicide. Stigma has been identified as a key risk factor for suicide among HIV-affected adolescents (Yang and Yang, 2013).

#### 2.2.2 Self-perception dimension: negative self-concept and identity confusion

Impaired self-perception starts when children take in their parents’ stigma about illness. This leads to feelings of low self-esteem, low self-efficacy, and confusion about their identity (Banerjee et al., 2010). In a study of Indian children, Das et al. Both HIV-infected and HIV-exposed uninfected children had lower self-esteem than the general pediatric norm. Also, self-esteem was negatively linked to parental stigma scores (Lin et al., 2011). Stage-specific manifestations vary across developmental periods. In the preschool years, children exhibit delayed development of self-efficacy, a lack of confidence in peer interactions, and low self-assessment of their abilities (Rafiei et al., 2023). During school age, kids who take on the role of “guardian of family secrets” often pull away from their friends. Deprived of positive self-feedback, they gradually develop a negative self-concept (Asadullah et al., 2017). In adolescence, identity confusion peaks. Adolescents face a dual conflict between their family identity—having HIV-positive parents—and their peer group identity. Some fully internalize the associated stigma, which can have irreversible effects on their self-identity in adulthood (Wanjala et al., 2023).

#### 2.2.3 Social adaptation dimension: lack of interpersonal trust and entrenched social avoidance

Impaired social adaptation comes from low interpersonal trust and strong social avoidance. This often results in weak peer relationships and delays in social skills (Zhang et al., 2010). Asadullah’s study shows that HIV-affected children exhibit significantly lower levels of social participation and peer interaction frequency compared to their peers, with a social avoidance rate 2.7 times higher than that of the general population (Zhang et al., 2010). Stage-specific manifestations include: in the preschool years, low willingness to interact with peers, passive withdrawal during group play, and excessive wariness toward strangers and unfamiliar environments (Peng, 2019), during the school-age period, they actively avoid group activities and deep interpersonal interactions, deliberately conceal their family circumstances, refuse to invite classmates home, and struggle to establish stable peer relationships due to a lack of interpersonal trust (Ameri et al., 2024), during adolescence, their social circles become closed off, sometimes leading to complete social isolation, with severe obstacles in establishing intimate relationships; they fear rejection by partners due to their family’s history of infection, and this avoidance pattern continues to affect social adaptation into adulthood (Wanjala et al., 2023).

#### 2.2.4 Behavioral dimension: high prevalence of internalizing and externalizing problem behaviors

Impairments in the behavioral dimension manifest as a high prevalence of both internalizing and externalizing problem behaviors. Internalizing problems include issues like academic struggles, withdrawal, and somatic symptoms. Externalizing problems mainly involve aggressive behavior, rule-breaking, and risk-taking actions (Cederbaum et al., 2015). Analyses confirm that the prevalence of behavioral problems among HIV-exposed but uninfected children ranges from 24.5 to 27.8%, showing no significant difference compared to infected children (Cederbaum et al., 2015). In preschool, children often show externalising problems like hyperactivity, aggression, and rule-breaking. These issues can disrupt eating and sleeping (Peng, 2019). As kids reach school age, academic troubles emerge. This includes inattention, truancy, and refusing to go to school. They may also experience physical symptoms like headaches and stomach pain without any medical cause (Dessie and Zewotir, 2024). In adolescence, risky behaviors become common. This includes substance abuse, risky sexual behavior, and antisocial actions. The risk of self-harm and suicide peaks, creating a harmful cycle (Chi and Li, 2013).

Existing evidence confirms that there is a clear dose–response relationship between parental stigma and children’s psychological harm. This relationship is moderated by multifaceted factors—including the child’s individual characteristics, family structure, and social environment—and exhibits significant population heterogeneity (Nelson et al., 2007).

#### 2.2.5 Core manifestations of the dose–response relationship

The dose–response relationship is primarily manifested across three dimensions: intensity, duration, and coping strategies. First, the intensity-dose effect. Parental internalized stigma is a key predictor. For every 1-standard-deviation rise in stigma scores, the risk of anxiety and depression in children goes up by 42 to 68% (Rzeszutek et al., 2021). Cohort studies confirm that the higher the level of parental co-stigma concerns, the lower the children’s interpersonal trust and the more severe their social avoidance (Sariah et al., 2016). Second, the cumulative effect of duration. Chronic stigma exposure in early childhood (ages 0–6) causes lasting psychological harm (Long, 2010). In contrast, short-term stigma during stressful times can be reversed if parents use positive coping strategies (Mwapasa et al., 2017). Third, the moderating effect of coping styles. When parents adopt positive coping strategies, the transmission effect is attenuated, and the slope of the dose–response relationship is flattened (Gao et al., 2018), conversely, negative coping amplifies the effect, such that even moderate levels of stigma exposure can lead to significant psychological harm in children (Wang et al., 2019).

#### 2.2.6 Key dimensions of population differences

Regarding gender, girls are more sensitive to impairments in the emotional dimension, and the dose–response relationship between parental stigma and girls’ internalizing problems (anxiety, depression) is more pronounced; for boys, the association is stronger with externalizing problems (aggression, rule-breaking) (Sariah et al., 2016). For preschool-aged children, the dose–response relationship only affects emotional and attachment dimensions. But for school-age kids, it covers all dimensions. The strongest effects are seen in adolescence (Lin et al., 2011). Children who know their parents are HIV-positive feel more psychological distress linked to parental stigma. This is especially true when the disclosure brings feelings of shame (Yehuda and Lehrner, 2018). Family structure and infection traits, along with disease stigma, create a steeper dose–response curve in families with two HIV-positive parents compared to single-parent families (Mwapasa et al., 2017). A mother’s stigma affects young children 2.1 to 2.8 times more than a father’s (Nabunya and Namuwonge, 2023). In low-income, low-education households, this relationship is even stronger because they lack coping resources (Xiong et al., 2022). Third, differences in the social environment. In rural areas and communities with high levels of stigma, the intergenerational transmission of parental disease-related shame is stronger (Nabunya et al., 2023); conversely, in families receiving adequate social support (formal medical support and informal support from relatives and friends), the dose–response relationship is significantly mitigated (Nabunya et al., 2023).

## 3 Core mechanisms and an integrated model of the intergenerational transmission of stigma among HIV-positive parents

### 3.1 Core mediating pathways in the intergenerational transmission of stigma

The stigma faced by HIV-positive parents affects their children’s psychological growth indirectly. This impact moves through various connections within the family system. These pathways act as “bridges” that pass disease-related stigma from parents to children. They are also key targets for efforts to stop this transmission. They primarily include four key pathways: family communication and disease disclosure, parent–child attachment and family functioning, parental emotions and parenting behaviors, and children’s cognition and internalization of stigma. Furthermore, these pathways are interconnected through a chain reaction effect.

#### 3.1.1 The mediating role of family communication and disclosure of illness

Family communication patterns and behaviors related to the disclosure of HIV infection serve as the most direct mediators of the intergenerational transmission of illness-related stigma. Goffman’s theory of stigma shows that “secret management” is key for those facing stigma. In HIV-positive families, this means certain communication rules. These rules then affect the cognitive and emotional growth of children (Goffman, 2009). The level of disease-related shame among HIV-positive parents is significantly negatively correlated with the openness of family communication. Parents who feel a lot of shame about disease often avoid talking about HIV. They may hide their infection status from their children. This creates a culture of secrecy in the family (Goffman, 2009). A study of 1,254 HIV-positive parents in Southwest China confirmed that the stronger the parents’ sense of disease-related stigma, the lower the rate of disclosure of their HIV status to their children. Furthermore, the more intense the feelings of shame and fear conveyed during the disclosure process, the significantly higher the risk of children developing symptoms of anxiety and depression (Qiao et al., 2015).

The logic of intergenerational transmission in this communication pattern manifests in two ways: First, when parents avoid talking about HIV, children miss out on learning. This can lead them to form negative views, like thinking “HIV equals shame and danger.” They often pick up these ideas from informal sources, linking their family’s HIV status to self-denial (Åsander et al., 2009). Second, when information is kept secret for a long time, trust between parents and children breaks down. Children may feel suspicious and anxious about their parents’ odd behavior. This pressure can turn into a shared experience of stigma (Das et al., 2017). A study of African HIV-positive parents residing in Sweden also found that only 13.6% (8 out of 59) of school-aged children were aware of their mothers’ HIV status. The low disclosure rate, due to parents feeling shame about their illness, puts children in a tough spot. They know there’s a family secret but cannot learn it. This situation raises their risk of social avoidance and low self-esteem (Åsander et al., 2009).

#### 3.1.2 The mediating role of parent–child attachment and family functioning

The quality of parent–child attachment and the integrity of family functioning serve as core emotional mediators in the intergenerational transmission of disease-related stigma. Their mechanism follows key ideas of family systems theory. This theory views the family as an interconnected whole. It also shows that parents’ mental states affect their children’s development through interactions (Olson et al., 2019). HIV-positive parents face stigma that harms their bond with their children and family life. First, high stigma leads to feelings of shame, anxiety, and depression. This makes it hard for them to respond to their children’s emotional needs in a caring and steady way. This prevents children from forming secure attachments. They miss out on a “secure base” for their psychological growth (Xiong et al., 2022). Also, stigma leads to problems like social isolation, marital conflict, and financial pressure. These issues weaken the family’s ability to solve problems and provide emotional support. They also disrupt how roles are divided, throwing the family system off balance (Qiao et al., 2015).

Existing empirical research has confirmed the mediating effects of parent–child attachment on family functioning. Studies have found that HIV-positive parents’ stigma indirectly increases the risk of psychological and behavioral problems in their children by reducing family cohesion and increasing parent–child conflict (Xing, 2020); longitudinal studies of adolescents with perinatal HIV infection have shown that insecure parent–child attachment fully mediates the relationship between parental stigma and children’s depressive symptoms, and this mediating effect is particularly pronounced during adolescence (Funck-Brentano et al., 2016). Impaired family functioning makes other issues worse. In dysfunctional families, children often copy their parents’ negative parenting. Also, family support is less likely to lessen the negative effects of disease disclosure (Ran et al., 2022).

#### 3.1.3 The mediating role of parental emotions and parenting behaviors

Parents’ emotional regulation patterns and parenting behaviors serve as key behavioral mediators in the intergenerational transmission of stigma. This transmission mechanism fits well with social learning theory. Children learn their parents’ emotional responses and behaviors by observing and imitating them (Bandura and Walters, 1977). The stigma experienced by HIV-positive parents directly shapes two types of maladaptive parenting behaviors: one is overprotection and excessive control. Parents worry their children might face discrimination. So, they limit their kids’ social activities and personal information. They might even interfere with their academic and life choices. This parenting style deprives children of the space to develop autonomy and reinforces their negative self-perception that “they are defective” (Rochat et al., 2016); The other is emotional neglect and parenting deficits. Emotional exhaustion from stigma can make parents withdraw. This can lead to them not responding well to their children’s needs. It may also result in parenting absence and a loss of control over behavior (Qiao et al., 2015).

At the same time, parents’ emotional regulation patterns are transmitted to their children through the process of “emotional socialization.” Parents who face a lot of disease-related stigma often use unhelpful coping methods. They might suppress their emotions or avoid dealing with the issue. Children learn to manage their emotions by watching and imitating others. This can slow down their ability to regulate emotions effectively (Huang et al., 2009). Research by Dong Liting confirmed that HIV-positive parents’ stigma is significantly positively correlated with anxiety and depressive symptoms, and that parents’ negative emotions directly affect the sensitivity of their parenting behaviors, thereby leading to emotional regulation difficulties and behavioral problems in their children (Huang et al., 2009); An intervention study by Rochat also demonstrated that when parents’ emotional regulation skills improved, the adaptiveness of their parenting behaviors significantly increased, and the incidence of psychological problems among their children decreased by 38% (Rochat et al., 2016).

#### 3.1.4 The mediating role of children’s cognition and internalization of stigma

Children’s thinking and the way they internalise stigma are key in passing down disease-related stigma. This shows how stigma changes from an outside influence to an internal belief (Corrigan and Watson, 2002). The stigma around HIV-positive parents affects children’s cognitive growth in two main ways. First, through cognitive modeling: Parents’ negative views and constant worry about discrimination influence their children. This can make kids think things like, “HIV infection is shameful” and “My family is different and inferior” (Ashry et al., 2017). Second, the experiential learning effect: the secondary discrimination and social exclusion children face due to their parents’ HIV status reinforce their identification with these negative cognitions, leading them to internalize external stigma as self-stigma (Qiao et al., 2015).

A study of 33 children with HIV-positive parents in Egypt found that these children had lower health-related quality of life (HRQOL) than those with HIV-negative parents. They also showed clear difficulties in psychological, family, and peer interactions. The core reason lies in their internalization of the stigmatizing belief that “they are excluded because of their parents’ infection” (Ashry et al., 2017). Children’s cognitive development affects how they internalize stigma. Younger kids often pick up stigma from their parents’ feelings and actions. In contrast, school-age and teenage children actively process stigma by comparing themselves to peers. This puts them at a greater risk of internalizing stigma (Corrigan and Watson, 2002). Research has found that among children who are aware of their parents’ HIV status, the correlation between their level of internalized stigma and their parents’ sense of shame is significantly higher than among children who are unaware, and this correlation is most pronounced among adolescents aged 10–16 (Qiao et al., 2015).

### 3.2 Key moderating factors in the intergenerational transmission of stigma

The intensity and direction of the effects of intergenerational transmission of stigma among HIV-positive parents are not fixed but are moderated by multiple factors at the individual, family, and societal levels. These moderating factors decide if disease stigma affects children’s psychological development. They do this by changing how well mediating pathways work. Understanding how these factors act is key for effective interventions (see Figure 1).

**Figure 1:**
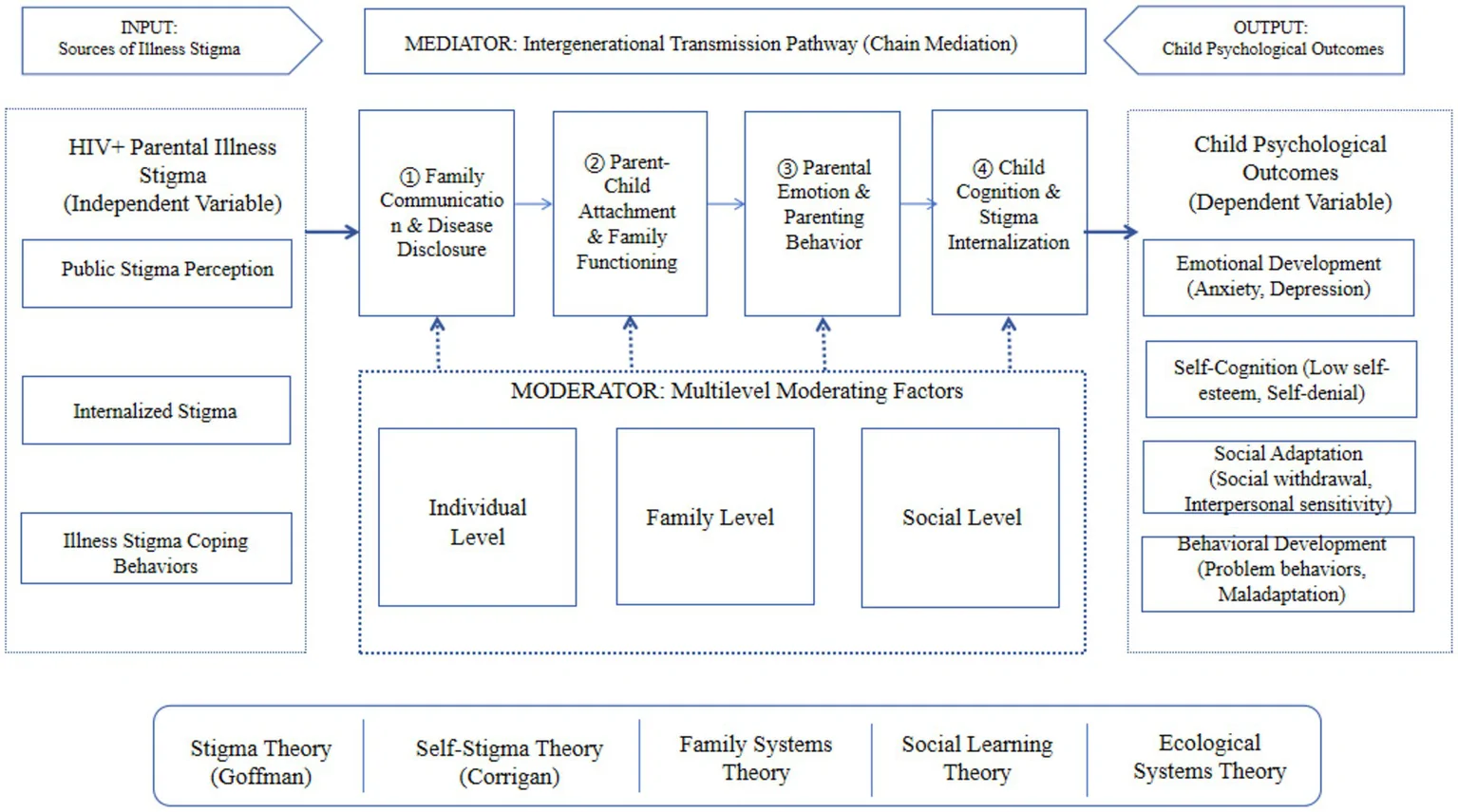
Integrated mechanism model for intergenerational transmission of stigma among HIV-positive parents.

#### 3.2.1 Moderating factors at the individual level

Moderating factors at the individual level primarily focus on the personal characteristics of parents and children. On the parental dimension, antiretroviral therapy (ART) adherence and disease control status are core moderating variables: A multicenter cohort study by Zerdali confirmed that HIV-positive mothers who adhered to ART during pregnancy and maintained a controlled viral load at delivery exhibited significantly lower levels of disease-related stigma compared to those with irregular treatment, and exhibited weaker psychological transmission effects on their children (Zerdali et al., 2023); This finding aligns with the research, which indicated that parents with well-controlled disease are more likely to develop a positive disease identity, thereby reducing negative emotions and maladaptive parenting behaviors (Ran et al., 2022). Also, the type of disease-related stigma and coping strategies matter. Parents with high internalized stigma often influence their children through emotions. In contrast, parents with high perceived stigma are less likely to affect their kids, thanks to strong social support (Mak et al., 2017).

In the child dimension, age and cognitive development play key roles. Preschool children, with limited cognitive skills, see parental disease stigma mainly through their attachment to parents. This leads to weaker effects. In contrast, adolescents seek self-identity, care more about social evaluation, and have better cognitive skills. As a result, they experience much stronger effects of disease stigma transmission (Wanjala et al., 2024). Research confirms that adolescents aged 10–18 internalize parental illness-related stigma significantly more than children under 10, and girls are more sensitive to emotional transmission pathways than boys (Mo et al., 2015). Children’s psychological resilience and coping skills act as protective factors. Kids with strong resilience can better understand their parents’ illness-related stigma. This helps them avoid internalizing self-stigma. Even in a family with high stigma levels, their risk of psychological issues is much lower (Hu et al., 2021).

#### 3.2.2 Family-level moderating factors

Family-level moderating factors influence the efficiency of the mediating pathways by altering the transmission of disease-related stigma. The level of family social support is a core protective factor: in HIV-positive families that receive support from spouses, relatives, and the community, parents exhibit lower levels of disease-related stigma, family functioning is more intact, and parent–child communication is more open, significantly weakening the intergenerational transmission of disease-related stigma (Chu and Wenbo, 2014). Conversely, in families lacking social support, parents’ negative emotions and parenting pressures cannot be buffered, amplifying the transmission effect (Ran et al., 2022). Parental educational attainment and family economic status also play significant moderating roles: Ashry found that parental educational attainment is an independent predictor of children’s HRQOL; parents with higher educational attainment are more likely to adopt scientific communication and parenting approaches, thereby reducing the transmission of disease-related stigma (Ran et al., 2022); conversely, low-income families often face the dual burden of survival pressures and disease-related stigma, resulting in a stronger transmission effect (Chu and Wenbo, 2014).

The moderating role of family culture and communication patterns is particularly pronounced: in families strongly influenced by traditional notions such as “family shame must not be aired in public,” parents’ disease-related stigma is more likely to manifest as communication avoidance and secret-keeping, thereby reinforcing the transmission effect (Xing, 2020); conversely, open family communication patterns can significantly buffer this process, allowing parents—even those with some degree of disease-related stigma—to reduce their children’s suspicion and internalization of stigma through candid dialogue (Qiao et al., 2015). Family structure impacts how HIV is transmitted. In families with both parents HIV-positive, stigma builds up, raising transmission risk. On the other hand, support from an HIV-negative spouse can lessen stigma’s impact, which helps lower transmission rates (Chu and Wenbo, 2014).

#### 3.2.3 Moderating factors at the societal level

Societal factors shape the “external environment” for disease stigma. They influence how stigma passes between generations. This happens by affecting parental views on disease and how families interact. Community inclusivity and HIV-related discrimination are key factors. When anti-stigma campaigns work well and communities accept people living with HIV, parents feel less stigma. This leads to less social isolation, better family functioning, and weaker disease-related stigma (Bharat, 2011). Conversely, communities with high levels of discrimination exacerbate parents’ disease-related stigma and social avoidance, creating a vicious cycle of “social discrimination → parental disease-related stigma → family isolation → children’s internalization of stigma” (Qiao et al., 2015).

Access to medical and social services also plays a crucial moderating role: A study by Fabusoro indicated that in HIV-positive families with access to standard ART treatment, regular psychological support, and case management services, parents’ disease-related shame and psychological stress were significantly reduced, and the negative impact transmitted to their children was minimized (Fabusoro and Mejia, 2021); The implementation of China’s “Four Frees and One Care” policy has also confirmed that comprehensive medical coverage and social support services can effectively reduce parents’ illness-related shame and provide buffering resources for families (Chu and Wenbo, 2014). Media coverage and health education are important. Good reporting on HIV can lower stigma and create a welcoming space for HIV-positive families (Åsander et al., 2009). On the other hand, negative and moralising reports can increase shame for parents and worsen issues passed down through generations (Mo et al., 2015).

### 3.3 An integrated theoretical model of the intergenerational transmission of stigma

#### 3.3.1 Core structure and theoretical Foundation of the Model

The integrated model has four main modules that connect in a closed loop. First, the three-dimensional input module focuses on independent variables. It centres on the stigma faced by HIV-positive parents. This module breaks down into three dimensions: “perceived public stigma,” “internalized stigma,” and “stigma coping behaviors.” The theoretical foundation of this module draws from Goffman’s theory of stigma (Goffman, 2009) and Corrigan’s self-stigma model (Corrigan and Watson, 2002), emphasizing that disease-related shame is the result of the combined effects of social construction and individual internalization, and that different dimensions of disease-related shame are transmitted through distinct pathways. Public stigma mainly affects family functioning through social interactions. Internalised stigma spreads through emotions and thoughts. Coping with illness-related shame directly influences family communication and parenting styles (Qiao et al., 2015).

Second, the four-level mediation module (mediating variables) comprises four progressive mediating links: “family communication and disclosure → parent-child attachment and family functioning → parental emotions and parenting behaviors → children’s cognition and internalization of stigma.” This module is grounded in family systems theory (Olson et al., 2019) and social learning theory (Bandura and Walters, 1977), emphasizing that the intergenerational transmission of illness-related stigma is achieved gradually through multiple interactions within the family system, and that the mediating effects of the preceding stage directly influence the transmission efficiency of the subsequent stage. Parents’ illness-related stigma impacts family communication. This disrupts parent–child attachment and is then passed to children through parenting behaviors. Ultimately, this leads to children internalizing the stigma (Rochat et al., 2016).

Third, the multi-level moderation module (moderating variables): This encompasses moderating factors at the individual, family, and societal levels. At the individual level, it looks at how well parents follow treatment and how resilient children are. At the family level, it focuses on social support and communication. At the societal level, it examines community inclusivity and access to services. Based on ecosystem theory (Rochat et al., 2016), this module emphasizes that the intergenerational transmission of stigma is a process embedded within multiple ecosystems; moderating factors at different levels influence the final transmission effect by altering the transmission efficiency of mediating pathways (Zerdali et al., 2023).

Fourth, the Multidimensional Output Module (Dependent Variables): This module centers on children’s psychological development outcomes, specifically encompassing four dimensions: emotional development (anxiety, depression), self-perception (low self-esteem, self-denial), social adaptation (social avoidance, interpersonal sensitivity), and behavioral development (problem behaviors). This module is based on the multidimensional theory of psychological development. It highlights how the intergenerational transfer of disease-related stigma affects children in many ways. Different pathways lead to different outcomes (Ashry et al., 2017).

#### 3.3.2 Operational logic and theoretical value of the model

The main idea of this model is simple: the stigma faced by HIV-positive parents affects their children’s psychological growth in various ways. This happens through a four-level mediation process. Also, factors from individual, family, and society can change how strong or weak this effect is at different stages of the process. Specifically, in the “input → mediation” stage, factors such as parental ART adherence, family social support, and community inclusivity determine whether disease-related stigma will translate into family communication barriers and functional imbalance; in the “mediation → output” stage, factors such as the child’s age, psychological resilience, and parental educational level determine whether negative changes at the family level will ultimately result in impaired psychological development in the child (Ran et al., 2022).

Theoretically, this model achieves three major breakthroughs: First, it combines different theoretical perspectives. This helps to overcome the limits of single-theory explanations and shows how disease stigma passes through generations. Second, it clarifies how mediating pathways are connected and identifies the targets of moderating factors. This fills a gap in past research about transmission boundary conditions. Third, it considers unique factors in HIV-positive families, like treatment status and family roles. This makes the theory more specific and applicable (Qiao et al., 2015). The model gives clear advice for improving intervention strategies. Input Stage: To reduce transmission sources, we can ease parental disease-related stigma and boost treatment adherence. Input Stage: To reduce transmission sources, we can ease parental disease-related stigma and boost treatment adherence. Mediation Stage: We can disrupt transmission pathways by enhancing family communication and fixing parent–child bonds. Moderation Stage: Strengthening social support and improving children’s psychological resilience can help lessen transmission effects (Rochat et al., 2016).

## 4 Interventions, outcomes, and population suitability regarding the intergenerational transmission of stigma among HIV-positive parents

### 4.1 Core categories and key characteristics of interventions

Based on ecosystem theory and family systems theory, and considering the mechanisms underlying the intergenerational transmission of disease stigma, existing interventions can be categorized into four core types according to their target populations and intervention foci: individual parent-targeted interventions, family systems-integrated interventions, child-protective interventions, and socio-ecological structural interventions. These four categories of interventions complement one another and build upon each other, forming a comprehensive intervention framework that spans from source control to environmental optimization. Each category possesses clear objectives and implementation characteristics.

#### 4.1.1 Parent-targeted interventions

This category of interventions serves as a source-level measure to interrupt the intergenerational transmission of disease-related stigma. Its main goal is to lower internalized stigma among HIV-positive parents. It aims to help them cope with stigma and manage their emotions. This way, we can tackle the root causes of stigma passed on to their children. Its core traits focus on parents only. It includes three main modules: cognitive restructuring, emotional management, and stigma coping skills. The program uses flexible, short-term formats, fitting for parent groups at various stages of infection and from different backgrounds.

Mainstream intervention techniques include cognitive behavioral therapy (CBT), acceptance and commitment therapy (ACT), and stigma coping skills training. Cognitive behavioral therapy lowers stigma scores tied to personal shame and poor self-image. It does this by changing parents’ negative thoughts and biases about HIV infection. A meta-analysis incorporating 32 RCTs confirmed that this therapy achieved an effect size of SMD = −0.58 (95% CI: −0.72 to −0.44) in improving internalized stigma among people living with HIV, and it significantly alleviated symptoms of anxiety and depression (Dessie and Zewotir, 2024). Acceptance and Commitment Therapy (ACT) helps parents accept their disease identity. It also reduces avoidance of experiences. This makes it great for groups facing internalised stigma and strong self-denial (He et al., 2022). Stigma coping skills training uses methods such as scenario simulation and role-playing to enhance parents’ ability to cope with external discrimination and engage in selective disclosure. A study by Qiao involving 1,254 HIV-positive parents in southwest China showed that parents who received this training exhibited a 37.2% increase in their willingness to disclose their status, along with significant improvements in mental health (Qiao et al., 2015). Mindfulness therapy helps reduce stigma-related rumination by increasing present-moment awareness. Its long-term effects are especially strong for parents with chronic mood disorders (He et al., 2022).

#### 4.1.2 Family systems integration intervention

This type of intervention is a core measure for severing the transmission pathways of disease-related stigma within families. This approach helps prevent the spread of disease-related stigma across generations. Its main features are: treating the family as a single unit for intervention, encouraging parents and children to participate together, and focusing on improving communication between them. It also aims to reshape disease narratives and boost family cohesion. It is the only intervention model capable of simultaneously addressing the source, pathways, and susceptible targets of intergenerational transmission.

Mainstream intervention models include family cognitive-behavioral therapy, parent–child communication training, and family narrative intervention. An intervention developed in South Africa serves as a typical example. Through six family sessions covering HIV disclosure guidance, health communication skills training, and custody planning, this intervention demonstrated in a randomized controlled trial that the rate of HIV disclosure by mothers to their children in the intervention group increased from 23.1 to 68.4%. Additionally, the level of open communication within families significantly improved, and the incidence of emotional problems among children decreased by 38% (Rochat et al., 2016). Research on HIV-positive families in rural China shows that family systems interventions can lower parents’ stigma scores by 31.2%. They also reduce parent–child conflict by 45.8%. These effects stay stable over a 12-month follow-up (Ran et al., 2022). Family narrative intervention helps family members share their life stories about the illness. It also works to replace negative ideas about the disease. This approach is especially good for families with teenage children. Research shows that this intervention can increase the proportion of open communication regarding the illness within the family by 62% and significantly reduce children’s feelings of shame regarding their parents’ illness status (Rochat et al., 2016). Parent–child communication training uses structured activities to tackle the “illness taboo” in families. It helps reduce the unspoken shame linked to illness. This training is especially helpful for families with preschool and school-aged children (Ran et al., 2022).

#### 4.1.3 Child-protective interventions

This type of intervention is a key measure for mitigating the susceptibility to intergenerational transmission of disease-related stigma. Its core objective is to enhance children’s psychological resilience and resistance to stigma, reduce the negative impact of parental stigma on their psychological development, and prevent the internalization of associated stigma and identity crises. Their main focus is on children’s developmental needs. The intervention content must match their age and cognitive level. It is often carried out in schools, communities, or child welfare agencies. The aim is to provide emotional support, cognitive education, and help children develop social skills.

Based on the intervention setting and content, these can be divided into three categories: psychological resilience-building interventions, peer support interventions, and cognitive education interventions. Psychological resilience interventions use group counseling and individual counseling to alleviate children’s anxiety and depression and enhance their self-esteem. A systematic review of children affected by HIV/AIDS in China showed that the effect size for improving depressive symptoms reached SMD = −0.59 (95% CI: −0.77 to −0.41) (Lin et al., 2011). Peer support interventions alleviate children’s feelings of loneliness and social withdrawal through the formation of peer groups and experience-sharing sessions. Research in Egypt confirmed that children participating in peer support groups scored significantly higher on all dimensions of health-related quality of life (HRQOL) compared to the control group, with particularly notable improvements in the dimension of interaction with family and friends (Ashry et al., 2017). Cognitive education interventions help children understand HIV better. They correct misconceptions and reduce feelings of shame by sharing age-appropriate scientific facts. This approach is highly effective among young children, but care must be taken to avoid overemphasizing the “special nature” of the disease, which could inadvertently reinforce stigma (Yuan and Zhang, 2017).

#### 4.1.4 Socio-ecological structural interventions

This type of intervention serves as a foundational measure to weaken the external reinforcing effects of intergenerational transmission of disease-related stigma. The main goal is to break down societal stigma, create a welcoming space for HIV-positive families, and lessen the factors that pass on stigma across generations. Their main traits are working with many stakeholders and covering various settings. These interventions have long-lasting effects and aim to be inclusive. They focus on four key areas: anti-discrimination in healthcare, community inclusion, media communication, and policy safeguards.

Current intervention practices primarily encompass four categories: First, anti-discrimination interventions in medical institutions, which reduce occupational discrimination in healthcare settings by providing HIV knowledge and anti-discrimination training to healthcare workers. A meta-analysis in China indicates that such training can reduce healthcare workers’ discriminatory attitudes toward people living with HIV by 22.7% and decrease the rate of refusal of medical services by 38.4% (Qiao et al., 2015); Second, community-based supportive interventions aim to reduce neighborhood discrimination through anti-stigma campaigns and the development of community support networks. Research in Tanzania indicates that peer support at the community level can reduce HIV-positive parents’ sense of social isolation by 42%, thereby mitigating the transmission of disease-related shame to their children (Gichane et al., 2018); Third, media-based science communication interventions: by presenting objective narratives of the disease, these efforts correct public stereotypes and dismantle moralizing metaphors. Research shows that sharing the life stories of people with HIV boosts public empathy and lowers stigma. Also, policy and legal protections are vital (Åsander et al., 2009). China’s “Four Frees and One Care” policy has enhanced support for HIV-positive families by safeguarding their rights and reducing structural stigma (Chu and Wenbo, 2014).

### 4.2 Systematic synthesis of the effects of different intervention models

#### 4.2.1 Evidence on the effects of parent-targeted interventions

Parent-targeted interventions are effective at reducing self-stigma and negative emotions. A meta-analysis by Rueda showed that cognitive behavioral therapy significantly reduced the total internalized stigma score among people living with HIV (SMD = −0.58, 95% CI: −0.72 to −0.44), with the most pronounced improvements observed in the dimensions of personal shame and negative self-image. It also significantly reduced anxiety and depressive symptoms, and the intervention effects remained stable during a 6-month follow-up (Dessie and Zewotir, 2024). Systematic reviews on mindfulness-based therapies confirm that their effect size for reducing feelings of stigma reaches SMD = −0.52 (95% CI: −0.68 to −0.36), and they outperform traditional cognitive interventions in enhancing emotional regulation abilities (He et al., 2022). Studies on middle-aged and elderly people living with HIV in China also indicate that Acceptance and Commitment Therapy (ACT) can reduce internalized stigma scores by 28.6% and significantly improve emotional regulation abilities (Zhang, 2011).

However, these interventions have clear limits. Individual efforts can only help parents improve their own condition. They do not directly stop transmission pathways within the family. Also, their effects on children’s psychological development are minimal. Multiple long-term follow-up studies have shown that for parents who received only individual interventions, there was no significant interruption in the intergenerational transmission of disease-related stigma to their children (Ran et al., 2022).

#### 4.2.2 Evidence on the effectiveness of family systemic integration interventions

Family systemic integration interventions are the best way to break the cycle of disease-related stigma. They help reduce stigma in parents, improve family dynamics, and protect children’s mental health. A systematic review incorporating 24 family intervention studies showed that the effect size for improving parents’ internalized stigma reached SMD = −0.61 (95% CI: −0.78 to −0.44), while simultaneously reducing children’s anxiety and depression symptoms by 47% and lowering the risk of internalizing stigma by 52% (Rochat et al., 2016). A study by Li focused on HIV-positive families in rural China. It found that family interventions can lower parents’ stigma scores by 31.2%. They also improve parent–child communication quality by 45.8% and boost children’s psychological resilience scores by 28.7%. These effects stayed stable during a 12-month follow-up (Ran et al., 2022).

#### 4.2.3 Evidence on the effectiveness of child-protective interventions

Child-protective interventions have a clear effect on improving the mental health and social adaptation of affected children. A systematic review of children affected by HIV/AIDS in China showed that psychological resilience interventions can significantly reduce anxiety (SMD = −0.63, 95% CI: −0.81 to −0.45) and depressive symptoms (SMD = −0.59, 95% CI: −0.77 to −0.41), while improving self-esteem and social adaptation (Lin et al., 2011). Peer support interventions can lower feelings of loneliness in children by 32%. They also reduce social avoidance behaviors by 28%. These effects are stronger in kids aged 6 to 12 (Yuan and Zhang, 2017). Cognitive education interventions can help correct children’s misunderstandings about HIV. They also reduce feelings of shame about the family’s disease status. A study in Egypt showed that children who received cognitive education had significantly higher HRQOL scores than the control group, particularly in the psychological and social dimensions (Ashry et al., 2017).

The main issue with these interventions is that they cannot stop disease-related stigma from being passed down through families. If parents’ stigma is not handled well, it will continue to affect the family and reduce the intervention’s success. Research indicates that in families with high parental stigma, the long-term effectiveness rate of interventions targeting only the child is less than 40% (Ran et al., 2022). Also, these measures are mostly “remedial.” This makes proactive prevention hard to achieve. Their fit for adolescent children relies on how the intervention content is designed for specific ages (Wanjala et al., 2024).

#### 4.2.4 Evidence on the effectiveness of socio-ecological structural interventions

The long-term effectiveness of socio-ecological structural interventions in reducing social stigma and improving the living conditions of HIV-positive families has been validated globally. A meta-analysis of anti-discrimination interventions in Chinese medical institutions shows that training for healthcare workers can reduce the stigma experienced by people living with HIV when seeking medical care by 38.4%, thereby lowering the level of internalized stigma among parents (Qiao et al., 2015). Community anti-stigma awareness campaigns and community support interventions can reduce discriminatory attitudes toward people living with HIV among community residents by 30% and enhance the community integration of HIV-positive families (Rochat et al., 2016). Media narrative interventions can significantly reduce public stigmatizing attitudes toward AIDS, increase empathy, and create a favorable external environment for family interventions (Åsander et al., 2009).

However, these interventions have delayed and indirect effects. Benefits from structural changes for individuals and families take 1–2 years to show up. This delay makes quick interruption impossible (Prunus salicina and Wang, 2016). Additionally, structural interventions alone cannot precisely target transmission processes within families; they must be combined with micro-level interventions to achieve maximum effectiveness.

### 4.3 Key moderating factors affecting intervention outcomes and the current status of localization

#### 4.3.1 Key moderating factors affecting intervention outcomes

At the individual level, the personal characteristics of parents and children serve as foundational moderating variables for intervention outcomes. On the parental dimension, disease stage and treatment adherence significantly influence intervention response: parents in the asymptomatic stage with high treatment adherence are more receptive to psychological interventions and show significant improvement in internalized stigma (Qiao et al., 2015); in contrast, parents in the AIDS stage with opportunistic infections focus their core needs on physiological treatment, and intervention participation rates are less than 20% (Zhou et al., 2021). Parental stigma affects responses to treatment. Parents who feel strong internal stigma benefit more from cognitive-behavioral interventions. In contrast, those facing high perceived stigma do better with social support and structural interventions (Dessie and Zewotir, 2024).

On the child dimension, age and cognitive development level determine intervention suitability: preschoolers are best suited for parent–child play and attachment-enhancing interventions; school-age children for school-family collaborative interventions; and adolescents for peer support and family narrative interventions (Wanjala et al., 2024). A study by Mo confirmed that adolescents aged 10–18 showed significantly higher participation in peer support interventions than younger children, whereas interventions targeting younger children are prone to triggering adolescent rebelliousness (Mo et al., 2015). Children’s awareness of their parents’ illness affects intervention results. For kids who get age-appropriate information, family communication interventions are 40% more effective. On the other hand, when parents hide the illness, these interventions have a hard time accessing key communication pathways (Qiao et al., 2015).

Regarding moderating factors at the family level, family system characteristics determine intervention participation and long-term outcomes. Family functioning and social support are key factors. In families with strong bonds and stable support from relatives and friends, the dropout rate is under 10%, and long-term outcomes remain stable (Ran et al., 2022). But in families with weak ties and little social support, the dropout rate is over 50% (Gichane et al., 2018). Family economic status also significantly moderates outcomes: in low-income families, parental stigma is deeply intertwined with survival anxiety; psychological interventions alone have limited effectiveness and must be combined with financial assistance and social security (Chu and Wenbo, 2014).

The moderating role of family culture and communication patterns is prominent: in families with a strong belief that “family matters should not be aired in public,” acceptance of open communication training is low, requiring the use of gradual narrative interventions (Xing, 2020); whereas in ethnic minority families, intervention models incorporating ethnic customs demonstrate significantly higher adaptability than Western-style interventions (Wang, 2021). Research in the Liangshan Yi region shows that family interventions with ethnic cultural elements increased participation rates to 75%. This is much higher than traditional interventions, which had a rate of 35% (Rochat et al., 2016).

Regarding moderating factors at the social structural level, the macro-social environment determines the accessibility and sustainability of interventions. Access to local resources is a key factor. In urban areas with strong HIV prevention systems, there are plenty of resources. This makes it easier to achieve lasting outcomes (Ran et al., 2022). However, in remote rural areas, the lack of professional support limits efforts to basic awareness campaigns (Zhou et al., 2021). Community inclusivity and the involvement of social organizations significantly influence intervention outcomes: in communities with established HIV prevention organizations, HIV-positive families face less discrimination, and the empowering effects of structural interventions are more pronounced (Rochat et al., 2016).

Policy support systems also play a moderating role: robust anti-discrimination legislation and healthcare coverage can reduce parents’ perceived stigma and provide support for micro-level interventions (Chu and Wenbo, 2014); conversely, in regions where policies are poorly enforced, even when individual interventions are implemented, parents’ levels of stigma are prone to rebound due to ongoing experiences of discrimination (Qiao et al., 2015).

## 5 Discussion

### 5.1 Theoretical and clinical implications of key findings

This review systematically examines the core mechanisms, intervention outcomes, and suitability characteristics of the intergenerational transmission of disease-related stigma among HIV-positive parents. Its key findings enhance our understanding of how stigma passes between generations. They also give clear, evidence-based advice for clinical practice and public health. This creates a strong link between theory and practical use.

Theoretically, the core contribution of this study lies in the construction of an integrated theoretical model featuring “three-dimensional inputs—four-level mediation—multi-level moderation—multi-dimensional outputs,” which overcomes the limitations of previous studies that relied on single theoretical perspectives or single-pathway analyses. This model is based on ecosystem theory. It combines stigma theory, family systems theory, and social learning theory. It shows that the passing down of disease-related shame happens through complex interactions. These interactions involve individual psychology, family dynamics, and social structures. It’s not just about copying emotions or behaviors (Goffman, 2009; Fabusoro and Mejia, 2021). Specifically, the mapping of the four-level mediation pathway (family communication and disclosure → parent–child attachment and family functioning → parental emotions and parenting behaviors → children’s cognition and internalization of stigma) reveals the “chain effect” through which illness-related stigma is transmitted from parents to children, addressing the shortcomings of previous studies that offered fragmented discussions of the transmission process (Qiao et al., 2015; Xing, 2020); Furthermore, the identification of multi-level moderating factors at the individual, family, and societal levels clarifies the boundary conditions of intergenerational transmission, addressing the core question of “why transmission effects vary across families despite similar levels of disease-related stigma” (Zerdali et al., 2023; Mo et al., 2015). This study has added to the understanding of how stigma passes between generations. It does this by including key aspects of HIV infection, like treatment adherence and disease disclosure. By also considering family roles, the research broadens the theory of stigma transmission to chronic infectious diseases. This creates a useful framework for studying stigma in other conditions, such as mental illness and hepatitis (Goffman, 2009; Corrigan and Watson, 2002).

At the clinical and public health practice levels, the practical value of these core findings is reflected in three dimensions: intervention targets, strategy selection, and population suitability. First, clarifying mediating pathways highlights key targets for intervention. Family communication and disease disclosure are the first steps in transmission. So, training parents in disclosure skills and offering counselling on open communication should be a priority (Qiao et al., 2015; Rochat et al., 2016). On the other hand, children’s understanding and internalisation of stigma come later. Thus, we need targeted interventions that focus on cognitive restructuring and building psychological resilience (Ashry et al., 2017). Second, integrating intervention outcomes gives evidence for choosing strategies. Family system interventions are best for breaking intergenerational cycles since they target multiple pathways (Rochat et al., 2016; Han et al., 2019). Parent-targeted interventions can lay the groundwork and work alongside family interventions to boost effectiveness (Armoon et al., 2021). Meanwhile, social-ecological structural interventions provide long-term support. They help create a better environment for micro-level interventions by reducing social stigma (Chu and Wenbo, 2014; Fabusoro and Mejia, 2021)^.^ Third, analyzing population-specific traits gives us guidelines for targeted interventions. For rural and ethnic minority families, we need to create modules that include local cultural aspects (Chu and Wenbo, 2014). For families with teens, we should enhance peer support and family narrative interventions (Wanjala et al., 2024; Mo et al., 2015). For low-income families, combining interventions with economic help and social security can reduce the impact of survival pressures on outcomes (Chu and Wenbo, 2014).

It should be noted that, although this review systematically retrieved literature from the global pool, it places greater weight on Chinese evidence in both mechanistic interpretation and intervention effect analysis. This emphasis is deliberate and rests on two considerations. China has a large population of HIV-positive parents—accounting for approximately 3.4% of the global total—with the epidemic concentrated in rural areas of the southwest and among ethnic minority communities (Joint United Nations Programme on HIV/AIDS [UNAIDS], 2025; National Center for AIDS/STD Control and Prevention, Chinese Center for Disease Control and Prevention, 2025). The epidemic mainly affects rural areas in the southwest and ethnic minority communities, where distinct regional and cultural traits exist. China’s “Four Frees and One Care” program provides valuable examples of structural interventions to reduce stigma. The lessons learned can be helpful for low and middle-income countries globally. Thus, this focus is not only practically warranted but also internationally relevant.

### 5.2 Dialogue with existing research and the innovative value of this review

This paper engages with existing research. It backs up key findings from earlier studies. It also makes new breakthroughs in three areas—research perspective, analytical framework, and practical implications. This work offers fresh insights for future research in the field. It confirms the central role of the family system in the intergenerational transmission of stigma, consistent with the findings of Rochat and Li, namely that family communication, parent–child attachment, and parenting behaviors are key mediators of this transmission. Additionally, this study found that comprehensive family system interventions yield the optimal outcomes, which aligns with the results of multiple global meta-analyses (Rochat et al., 2016; Armoon et al., 2021). Furthermore, the individual-level moderators identified in this review (e.g., parental treatment adherence, child age) are supported by the studies of Zerdali and Mo, further validating the stabilizing role of these factors in the transmission process.

The innovative value of this study shows in three main ways: First, it broadens the research perspective. Previous studies mainly looked at how stigma directly affects children or focused on single mediating pathways. In contrast, this review explores the transmission process within a nested ecosystem of “individual–family–society.” It clearly explains the chain-reaction effects of mediating pathways and the hierarchical roles of moderating factors. This approach offers a deeper understanding of the transmission mechanism (Qiao et al., 2015; Xing, 2020). Second, innovation in the analytical framework—the integrated theoretical model constructed clarifies the logical relationships and intervention targets among variables, resolving the issues of ambiguous variable relationships and unclear intervention targets in previous studies, and providing a standardized analytical framework for subsequent empirical research (Rochat et al., 2016; Han et al., 2019). Third, deepening practical focus—this review is the first to look closely at how cultural context and resource access affect intervention outcomes. It highlights a key gap in current research, which often “prioritises outcomes over adaptability” (Wang, 2021). For example, addressing the cultural norm in rural China that “family matters should not be aired in public,” this review proposes a gradual narrative intervention model rather than directly adopting Western open communication training, thereby significantly enhancing the practical feasibility of interventions (Xing, 2020).

Furthermore, this review integrates domestic and international evidence. These studies are drawn from China, Western countries, and other regions across Asia and Africa. This broad geographical coverage allows the review to address the differences and commonalities across diverse cultural and regional contexts. For instance, studies from sub-Saharan Africa and South Asia tend to emphasize limited social support and structural stigma (Olson et al., 2019). By contrast, studies from Western contexts focus more on family communication and individual psychological traits (Åsander et al., 2009). This review incorporates these differences into the analysis of moderating factors, constructing a theoretical model that is globally applicable while accounting for local characteristics, thereby providing tailored guidance for interventions in regions with varying resource conditions.

### 5.3 Limitations of this review

Although this review strictly adhered to systematic review methodological standards and sought to ensure the comprehensiveness and reliability of the results, the following limitations exist and should be considered when interpreting the results and planning future research.

First, there are limitations regarding the scope of the search and study inclusion. This review looked at key Chinese and English databases. It did not include gray literature, like unpublished reports and conference abstracts, or studies in other languages. This may lead to publication and language bias (Armoon et al., 2021). Furthermore, some of the included studies employed cross-sectional designs (accounting for 38.9%), while only 12 were longitudinal studies. This makes it difficult to fully validate the causal relationship and long-term effects of the intergenerational transmission of disease stigma, potentially leading to imprecise identification of mediating pathways and moderating factors (Han et al., 2019).

Second, heterogeneity across the included studies poses a challenge to interpretation. The included studies spanned regions including Africa, Asia, the Americas, and Europe. Significant differences exist across these regions in terms of HIV epidemiology, sociocultural contexts, and resource accessibility, which may result in heterogeneity in the effect sizes of intervention outcomes and moderating factors (Zerdali et al., 2023). Although this review considered the moderating roles of region and culture in its analysis, it did not conduct quantitative heterogeneity analyses, which may affect the stability and generalizability of the results.

Third, research on specific populations remains insufficient. Few of the included studies (17 in total, accounting for 13.5%) targeted specific subgroups (e.g., families of migrant populations, ethnic minority families, and families with adolescent parents), resulting in an insufficient understanding of the transmission mechanisms and intervention suitability for these groups (Olson et al., 2019). Furthermore, existing research findings regarding the impact of children’s HIV status (infected vs. uninfected) on transmission effects are inconsistent, and this review was unable to fully clarify the scope of this influence (Wanjala et al., 2024).

Fourth, there are notable limitations in the evaluation of intervention effectiveness. Most existing intervention studies rely on self-report scales to assess outcomes (e.g., anxiety and depression scales, stigma scales), while objective outcome measures (e.g., academic performance, clinical psychological diagnoses) are rarely used, which may lead to an overestimation of intervention effectiveness (Wanjala et al., 2024; Han et al., 2019). Additionally, the follow-up periods in most intervention studies are short (62.3% had a follow-up of ≤6 months), and there is a lack of tracking of long-term effects, making it difficult to assess the sustainability of intervention effects and the risk of relapse (Rochat et al., 2016).

## 6 Conclusion

Through a systematic synthesis of global and local evidence, this review clarifies the core mechanisms, intervention outcomes, and contextual adaptability of intergenerational stigma transmission among HIV-positive parents. The transmission works in a cascading way. It starts with family communication and disclosure. Then, it moves to parent–child attachment and family functioning. Next, parental emotions and parenting behaviors come into play. Finally, it affects children’s cognitive processing and stigma internalization. Each stage is influenced by personal, family, and societal factors. Among the available intervention approaches, family-based integrated interventions demonstrate the greatest efficacy due to their capacity to disrupt multiple mediating pathways simultaneously; parent-targeted and child-protective interventions serve as complementary strategies, while socio-ecological structural interventions provide the necessary enabling environment, underscoring the need for a coordinated, multi-modal approach. Intervention effectiveness depends on cultural context, available resources, and population traits. This shows the need for local strategies that consider regional differences, family setups, and social support networks.

Looking ahead, future research should prioritize three directions. First, we need high-quality longitudinal cohort studies with data from multiple time points. These studies will help us establish causality, clarify the sequence of mediating pathways, and explain how moderating factors work. Second, greater research attention should be directed to underserved populations—including migrant families, ethnic minority groups, and adolescent parents—to uncover their unique transmission dynamics and intervention needs. Intervention science should shift to multi-system collaborative models. These models need to include family, medical, community, and policy elements. Also, we should improve evaluation frameworks. They should mix subjective and objective measures and extend follow-up times to check for long-term sustainability and relapse prevention.

In sum, the intergenerational transmission of stigma among HIV-positive parents, while complex, is amenable to intervention. Its effective disruption depends on sustained synergy among theoretical inquiry, clinical innovation, and public health policy. The model and intervention framework from this review provide a standard base for future research and practice. They aim to lower psychological risks for children in HIV-affected families and improve family health and well-being.
